# Patient‐reported outcomes following participation in a preoperative peer support programme for total knee replacement: A prospective observational cohort study

**DOI:** 10.1002/jeo2.70777

**Published:** 2026-06-04

**Authors:** Fraser Middleton, Jonathan R. A. Phillips

**Affiliations:** ^1^ Medical School University of Exeter Exeter UK; ^2^ Royal Devon University Healthcare NHS Foundation Trust Princess Elizabeth Orthopaedic Centre Exeter UK

**Keywords:** arthritis, patient satisfaction, peer support, preoperative education, total knee replacement

## Abstract

**Purpose:**

Total knee replacement (TKR) is a highly successful operation; however, a proportion of patients remain dissatisfied following surgery, often related to unmet preoperative expectations. In response, Arthritis UK developed a pilot peer support programme for patients awaiting joint replacement. This study evaluated short‐term changes in patient‐reported experience following participation in the programme among patients awaiting TKR.

**Methods:**

This prospective observational cohort study included patients scheduled for primary TKR who participated in a structured peer support programme delivered in small group sessions. Participants completed a 10‐item programme‐specific questionnaire rated on a 10‐point visual analogue scale immediately before peer support, and on average after the second session. A composite experience score was calculated using mean item scores when at least 80% of items were completed. Pre–post changes were analysed using paired statistical tests.

**Results:**

Seventy participants were included in the analysis. The composite experience score increased by 1.2 points (standard deviation [SD] = 1.3), from 5.7 (SD = 1.3) pre‐intervention to 6.9 (SD = 1.1) post‐intervention. Patients reported increases in perceived emotional support and confidence in knowing what to expect from the operation and recovery period. Improvements were also observed in satisfaction with management plans and perceived ability to manage health. No significant changes were observed in self‐reported preoperative anxiety or in the perceived impact of joint pain on daily life.

**Conclusion:**

Participation in the peer support programme was associated with short‐term improvements in overall patient‐reported experience, particularly knowledge, emotional support, perceived control over health and treatment‐related satisfaction. These findings suggest that peer support may complement standard preoperative education by influencing how patients understand and emotionally experience their surgical journey. Further controlled studies with longer follow‐up are required to determine causality and evaluate whether these short‐term changes are sustained and associated with post‐operative satisfaction.

**Level of Evidence:**

Level IV.

**Abbreviations**
TKR: total knee replacement
VAS: visual analogue scale

## INTRODUCTION

Demand for total knee replacement (TKR) is increasing across the United Kingdom (UK) [11]. Over 100,000 TKRs are currently performed each year in the UK, with this number set to increase by almost 40% by 2060 [11]. But despite the volume of surgeries performed, it is well understood that a group of patients feel dissatisfied with their results at the 1‐year follow‐up post‐surgery. This figure is estimated to be between 10% and 22% [2, 3, 9, 15].

Unmet preoperative expectations are often overly optimistic and thus a major cause for patient dissatisfaction [6, 10]. A pilot study of total joint replacement patients found that more than half the patients had higher expectations than their surgeon for their recovery [6]. This was driven by expectations of participating in high‐level activities and regaining an extreme range of motion, such as kneeling and squatting [6]. Patients with milder osteoarthritis (OA) are also more likely to be dissatisfied post‐surgery [3]. This is likely due to greater expectations of return of function. The need for preoperative patient education is therefore important to help patients develop realistic expectations for the surgery and recovery process. Individual patient factors can also play a role in dissatisfaction [3, 4]. Anxiety, depression and living alone are psychosocial predictive factors of greater dissatisfaction post‐operation [3]. Having a lower internal locus of control may also contribute to greater dissatisfaction [4].

Peer support refers to individuals with shared experiences meeting to exchange information and provide mutual support [8]. While peer support has been widely studied in mental health settings, where its effectiveness is well established [14], its application in surgical populations remains underexplored. The information‐sharing component of peer support may help patients develop realistic preoperative expectations, while opportunities for shared experience may foster social connection and emotional support.

In 2018, the charity Arthritis UK launched a pilot peer support programme, ‘Joint Understanding’, for patients in the UK awaiting joint replacement. Although the programme also included patients undergoing hip replacement, the present analysis focuses on those scheduled for primary TKR. Despite growing interest in peer‐led approaches within perioperative care, there is limited empirical evidence evaluating their short‐term impact on patient‐reported experience prior to TKR. It remains unclear which domains, such as knowledge, emotional support, control over health or treatment satisfaction, may be most responsive to peer support interventions.

The primary aim of this study was to evaluate changes in overall patient‐reported experience following participation in a preoperative peer support programme for individuals awaiting TKR. A secondary aim was to explore which specific domains of patient experience appeared most influenced by participation. It was hypothesised that peer support would be associated with improvements in knowledge, emotional support and treatment satisfaction, but not necessarily with changes in perceived pain burden, control over health or preoperative anxiety.

## METHODS

### Ethics statement

The peer support programme was delivered by Arthritis UK as part of routine service provision. Data were collected for programme evaluation purposes. All participants provided written informed consent for data collection.

### Patient selection

Patients were eligible for inclusion if they were adults scheduled to undergo primary TKR and were able to attend peer support sessions at a local venue and complete questionnaires in English. Patients were recruited from surgical waiting lists at selected centres in Exeter, Sheffield, Middlesbrough and Crewe. Invitation letters were sent to patients, and those who responded were invited to attend peer support meetings. Patients undergoing revision arthroplasty, non‐knee joint replacement, or those unable to provide informed consent were excluded. Data were collected between January 2018 and June 2019.

### Development of peer support training

To identify the information patients would find most important in the pre‐ and post‐operative period, individuals who had previously undergone TKR were surveyed and their perspectives collated. These findings informed the development of a structured information programme for participants (included in Appendix 1) and a dedicated training package for volunteer peer supporters. Peer supporters were trained to facilitate sessions with oversight from Arthritis UK staff.

### Peer support sessions

Participants attended small, informal group sessions facilitated by trained volunteer patients at local venues, scheduled between 1 and 6 months before surgery. Participants attended a mean of two sessions prior to completing the post‐session questionnaire. Trained patients shared their experiences of TKR and recovery using a structured format and facilitated discussion. Discussion topics included:

- Benefits and risks of knee replacement surgery
- Preoperative exercises to improve recovery
- Different types of knee replacement surgery
- Anaesthesia used in knee replacement surgery
- Practicalities of staying in hospital
- Managing post‐operative pain
- Getting around post‐operation, including driving, walking, climbing stairs and returning to work
- Daily life post‐operation, including sleeping, getting dressed, housework, cooking, using the toilet, bathing, and sex and relationships
- Recommended post‐operative rehabilitation exercises

### Patient‐reported questionnaire

A 10‐item patient‐reported questionnaire was administered by charity staff immediately before the first peer support session, and on average after the second session. Participants rated agreement with each statement on a 10‐point visual analogue scale. The questionnaire was developed by Arthritis UK with the input from trained patients to capture domains that were deemed relevant to peer support. Internal consistency of the questionnaire at baseline was moderate (Cronbach's *α* = 0.62; *n* = 70), showing the multidimensional nature of the items. Three items were phrased such that higher scores represented worse outcomes. These items were reverse‐scored prior to analysis so that higher scores consistently reflected more favourable patient‐reported results. The full questionnaire is included in Appendix 2.

### Handling of questionnaire completion

Participants who did not complete the baseline questionnaire were excluded from the analysis. Participants who did not return a post‐intervention questionnaire were considered lost to follow‐up and thus were not included in pre–post change analyses.

The primary outcome was an overall patient‐reported experience score. This was calculated as the mean of completed items when at least 80% of items (≥8 out of 10) were answered at both time points. Participants below this threshold were excluded from the composite analysis but remained eligible for item‐level analyses if they provided paired responses for individual questions. A sensitivity analysis was conducted, including only participants with complete responses to all questionnaire items at both time points.

### Statistical analysis

Data management and preliminary handling were performed using Microsoft Excel (Microsoft Corp.). Statistical analyses were conducted using R statistical software (R Foundation for Statistical Computing).

Composite scores for the patient‐reported experience score were calculated as the mean of questionnaire items, with reverse scoring applied where appropriate. Normality of composite change scores was assessed through visual inspection of histograms and *Q*–*Q* plots and was considered adequate for parametric testing. Pre‐ and post‐session composite scores were compared using paired *t* tests. Sensitivity analyses restricted to participants with complete questionnaire data were undertaken to assess the robustness of findings. For item‐level changes, paired analyses were conducted using participants who provided both pre‐ and post‐session responses for that question. These analyses were considered exploratory.

As this study evaluated a routinely delivered service, the sample size was determined by programme attendance during the study period. To contextualise study sensitivity, a post hoc detectable effect size was estimated. With 70 paired observations, the study had 80% power (*α* = 0.05) to detect a mean pre–post change of approximately 0.45 points on the 0–10 scale, corresponding to a small‐to‐moderate standardised effect size (Cohen's *d* ≈ 0.34).

## RESULTS

**Figure 1:**
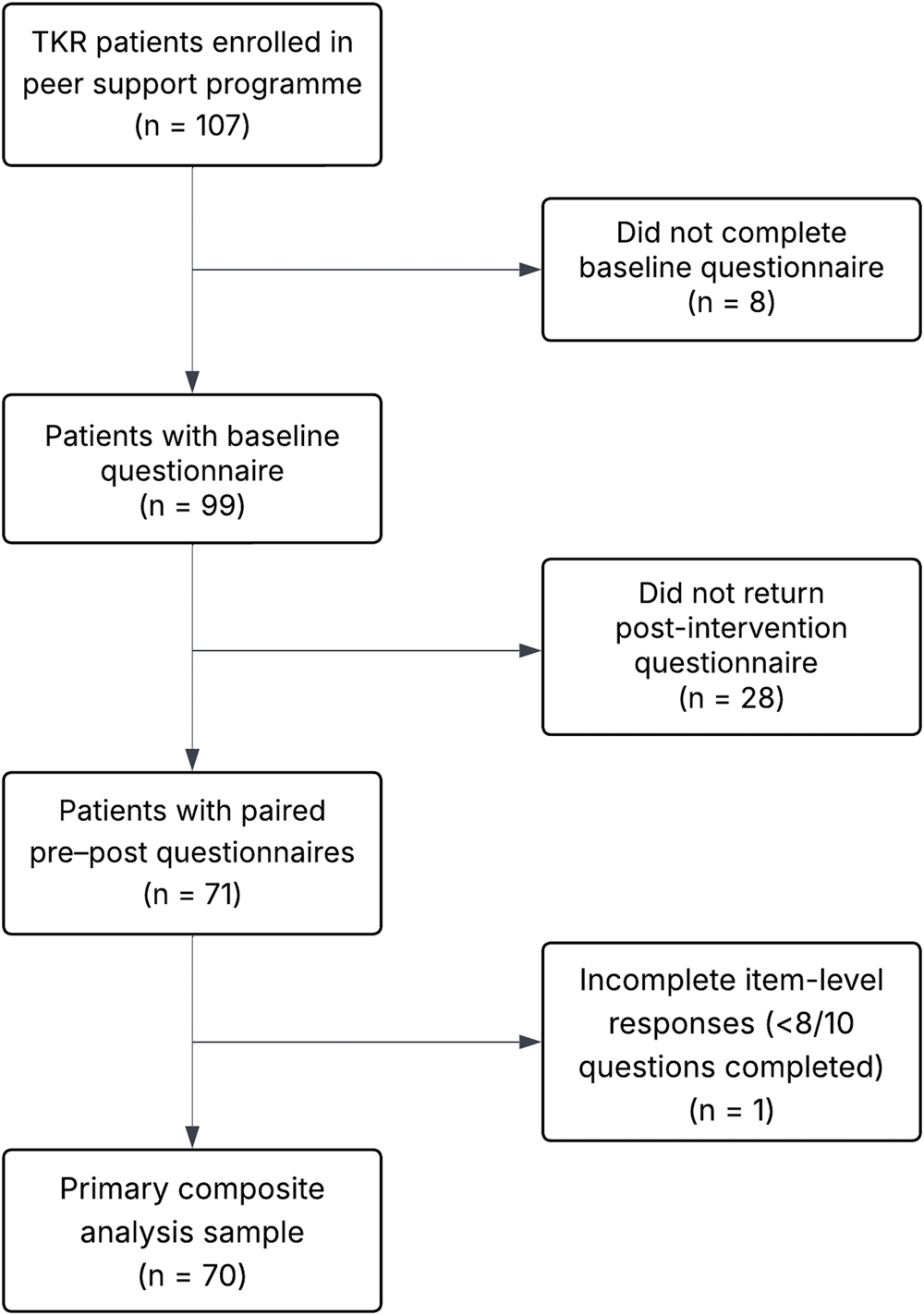
Flow diagram illustrating patient enrolment and inclusion for analysis. Of 107 patients scheduled for total knee replacement (TKR) who attended the peer support programme, 8 did not complete a baseline questionnaire, and 28 did not complete a follow‐up questionnaire. One additional patient did not meet eligibility criteria for calculation of the composite score (<80% questionnaire completion at both time points), resulting in 70 participants included in the final analysis.

A total of 107 TKR patients enroled in the peer support programme. Of these, eight did not complete a baseline questionnaire and were excluded from analysis. A further 28 participants did not return a post‐intervention questionnaire and were considered lost to follow‐up. Seventy participants were included in the primary composite analysis. Sixty‐five participants had complete questionnaire data and were included in sensitivity analyses. The patient enrolment process is shown in Figure 1.

### Primary outcome: Overall patient‐reported experience score

The overall patient‐reported experience score increased from 5.7 (standard deviation [SD] = 1.3) pre‐intervention to 6.9 (SD = 1.1) post‐intervention, representing a mean increase of 1.2 points (SD = 1.3). This change was statistically significant (*p* < 0.001). Sensitivity analyses restricted to participants with complete questionnaire data (*n* = 65) demonstrated similar changes in composite scores.

### Secondary outcomes: Item‐level analyses

Examination of individual questionnaire items suggested that improvements were most evident in domains related to knowledge and emotional support. Participants reported higher post‐intervention scores for feeling supported by others with similar experiences, feeling understood and confidence in knowing what to expect from the operation and recovery process. Improvements were also observed in satisfaction with pain management and perceived ability to manage one's health. In contrast, no significant changes were observed in the perceived impact of joint pain on daily life or anxiety related to surgery. Detailed item‐level results are presented in Table 1.

**Table 1:** Changes in patient‐reported questionnaire scores before and after peer support.

Question (higher scores indicate more favourable responses)^a^	*n*	Pre mean ± SD	Post mean ± SD	Mean change (95% CI)	*p*
I am satisfied with the pain relief I achieve for arthritis/joint pain with my current treatment plan/medication	69	4.8 ± 2.6	6.8 ± 2.4	2.1 (1.4–2.8)	*p* < 0.01*
I often feel like I cannot do anything myself to lessen the impact of arthritis on my life (reversed)	68	4.8 ± 2.9	4.0 ± 3.0	−0.8 (−1.5 to 0.0)	*p* = 0.058
I feel there is someone who understands what I'm going through	68	6.5 ± 2.5	8.3 ± 2.1	1.8 (1.2–2.4)	*p* < 0.01*
I have people around me who can support me	69	7.5 ± 2.6	8.2 ± 2.1	0.7 (0.1–1.2)	*p* < 0.05*
I am able to manage my health in ways that work for me	70	6.9 ± 2.2	7.6 ± 2.1	0.8 (0.3–1.3)	*p* < 0.01*
I feel anxious/worried about my operation (reversed)	71	4.5 ± 2.9	4.1 ± 2.7	−0.5 (−1.3 to 0.4)	*p* = 0.283
I have the information I need to know what to expect from the operation	71	6.4 ± 2.9	9.0 ± 1.4	2.6 (1.9–3.3)	*p* < 0.01*
I have the information I need to support my recovery after the operation	71	6.4 ± 2.9	8.9 ± 1.7	2.5 (1.7–3.2)	*p* < 0.01*
I have support from people who are going through the same experience	71	6.0 ± 3.0	8.3 ± 2.4	2.2 (1.4–3.0)	*p* < 0.01*
Overall, how would you rate the impact of arthritis/joint pain on your day‐to‐day life? 1 not at all, 10 very severe (reversed)*	69	3.1 ± 3.0	3.4 ± 2.9	0.3 (−0.5 to 1.1)	*p* = 0.443

*Note*: Higher scores indicate more favourable responses. Three items were reverse‐scored so that higher scores consistently reflected more favourable outcomes. Statistically significant differences were defined as *p* < 0.05.

Abbreviations: CI confidence interval; SD standard deviation.

^a^ Three items were reverse‐scored so that higher scores indicate more favourable outcomes.

* Statistically significant at *p* < 0.05.

## DISCUSSION

This study found that participation in a preoperative peer support programme was associated with short‐term improvements in patient‐reported experience among individuals awaiting TKR. The largest changes were observed in perceived knowledge of the surgery and recovery, emotional support, and confidence in managing health, while joint pain and preoperative anxiety showed little change. These findings suggest that peer support may primarily influence how patients understand and emotionally process their upcoming surgery rather than their perceptions of disease severity.

While peer support interventions have been widely explored in areas such as mental health [14], their application within joint replacement pathways remains relatively limited. Most perioperative preparation strategies in orthopaedics have focused on clinician‐led education and rehabilitation, with fewer studies evaluating structured peer‐led support programmes [12]. The present findings therefore contribute to an emerging area of research focused on understanding how peer interaction may influence patient experience prior to surgery.

The observed improvements in perceived knowledge about the operation and recovery process are highly relevant given the significant role that unrealistic preoperative expectations play in contributing towards post‐operative dissatisfaction [6, 10]. Many patients anticipate returning to participation in high‐level activities or restoration of the extreme range of motion, expectations that are often more optimistic than their surgeon's [6]. Interventions that support the development of realistic expectations may therefore be valuable in shaping how surgical outcomes are perceived. Traditional preoperative education in joint arthroplasty is typically clinician‐led and focused on providing structured information about the surgery and recovery period [12]. Peer support may offer something distinct: knowledge grounded in lived experience and delivered within a supportive social context. Theories of social comparison and observational learning suggest that individuals evaluate their own situation by referencing others with similar experiences [1, 5]. Hearing recovery narratives from peers who have undergone surgery may provide a benchmark for expected progress, helping patients contextualise challenges and individual variability in recovery. Peer modelling may enhance perceived self‐efficacy by demonstrating that others have successfully navigated the surgical journey [1]. This aligns with the observed increase in patients' confidence in managing their own health.

In addition to informational gains, interaction with peers who have undergone or are awaiting surgery may foster a sense of shared experience and understanding [8, 14]. Preparing for surgery can involve uncertainty and concern, and opportunities to discuss these feelings with others in similar circumstances may help normalise expectations and reduce feelings of isolation. Improvements in satisfaction with treatment plans and pain management may therefore reflect not only enhanced knowledge but also increased confidence and reassurance within the care pathway. When patients have a clearer sense of what to expect from recovery and feel supported in navigating this process, treatment strategies may appear more coherent and manageable.

In contrast to the improvements observed in knowledge, support, and treatment‐related satisfaction, perceptions of arthritis‐related symptom burden and preoperative anxiety showed little change. This pattern is consistent with the informational and supportive focus of the programme, which was not designed to modify symptom burden or directly target preoperative anxiety. Preoperative education and support interventions more broadly have shown limited and inconsistent effects on pain, function, and anxiety outcomes, suggesting that such domains may be influenced by factors beyond informational preparedness alone [12]. Importantly, pain and functional limitation in OA have been closely linked to the pathological progression of the disease, in which whole‐joint changes produce pain and functional limitation [7]. Research on psychological preparation before surgery has shown no clear consensus that brief informational or supportive approaches reliably reduce preoperative anxiety [13]. These findings therefore support the interpretation that peer support primarily influences how patients understand and experience their care rather than altering symptom experience itself.

This study has several limitations. As an evaluation of a pilot programme delivered in routine practice, detailed demographic and clinical characteristics were not systematically collected, limiting assessment of representativeness and preventing adjustment for potential confounding factors. However, participants were recruited through routine clinical pathways from existing surgical waiting lists and invited to attend a programme already delivered as part of standard service provision. Thus, the sample reflects real‐world service uptake rather than a research‐selected cohort, which may enhance ecological validity. The absence of a control group receiving standard care prevents causal inference, and thus observed changes may not reflect the peer support component alone. Outcomes were assessed immediately following the session, so the longevity of these changes and whether they influence post‐operative satisfaction or recovery are unknown. The questionnaire used was programme‐specific and not a validated psychometric instrument, and responses may have been influenced by social desirability or the immediate context of group discussion. Finally, incomplete questionnaire responses resulted in exclusion of some participants from sensitivity analyses, which may have introduced bias if non‐completion was associated with dissatisfaction or disengagement.

Future research should examine whether the short‐term observed benefits of peer support translate into differences in post‐operative satisfaction, recovery experience, or health‐related quality of life. Studies carried out with a control group would help distinguish the specific effects of peer support from non‐specific group or attention effects, while a mixed methods approach incorporating qualitative interviews could provide deeper insight into how patients interpret and apply information gained through peer interaction.

## CONCLUSION

Participation in the preoperative peer support programme was associated with short‐term improvements in patient‐reported understanding, emotional support, perceived control over health and treatment‐related satisfaction. These findings suggest that peer support may complement conventional preoperative education by influencing how patients interpret and emotionally experience the surgical journey. Further controlled studies with longer follow‐up are required to determine whether these changes are sustained and associated with post‐operative satisfaction.

## AUTHOR CONTRIBUTIONS

**Fraser Middleton**: Conceptualisation; data curation; formal analysis; writing—original draft; writing—review and editing. **Jonathan R. A. Phillips**: Resources; writing—review and editing.

## CONFLICT OF INTEREST STATEMENT

The authors declare no conflicts of interest.

## ETHICS STATEMENT

The peer support programme was delivered by Arthritis UK as part of routine service provision. Data were collected for programme evaluation purposes. All participants provided written informed consent for data collection.

## Supporting information

Supporting File 1.

Supporting File 2.

## Data Availability

The data that support the findings of this study are available on request from the corresponding author. The data are not publicly available due to privacy or ethical restrictions.
