# Supplementary figures and images for: Patient‐reported outcomes following participation in a preoperative peer support programme for total knee replacement: A prospective observational cohort study
Source: J Exp Orthop. 2026 Jun 4;13(2):e70777. doi: 10.1002/jeo2.70777 (PMC13288369; doi:10.1002/jeo2.70777)

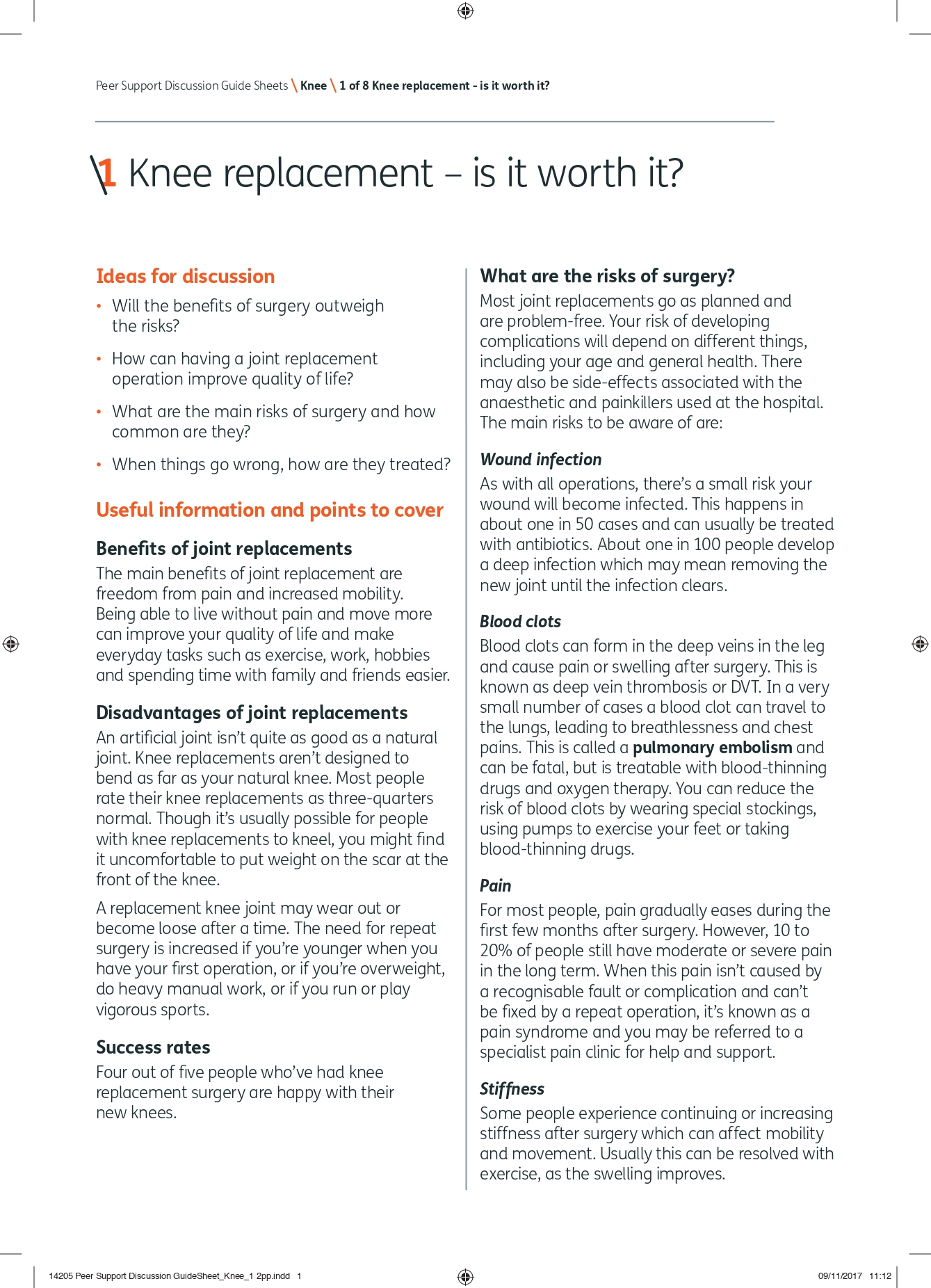

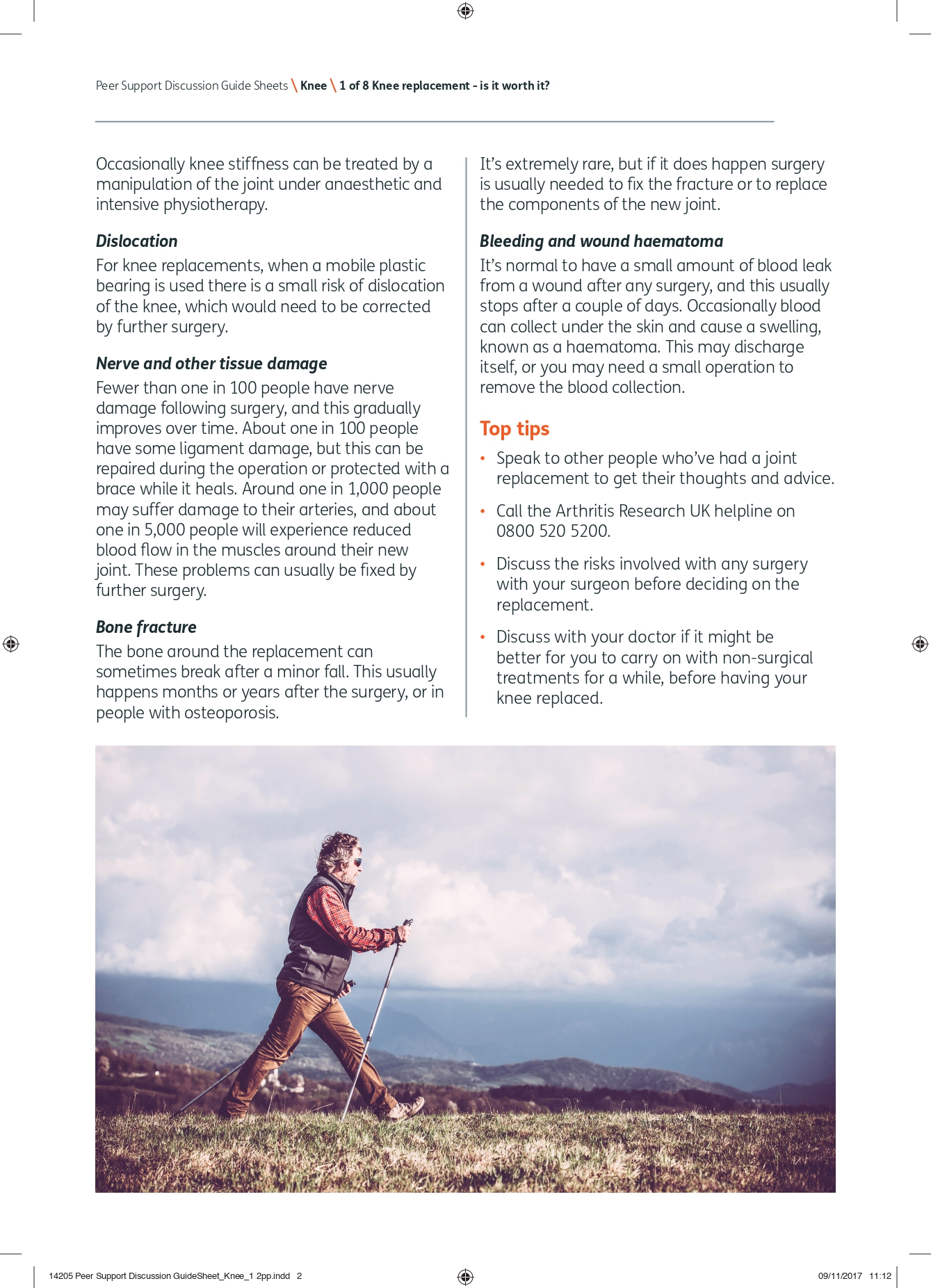

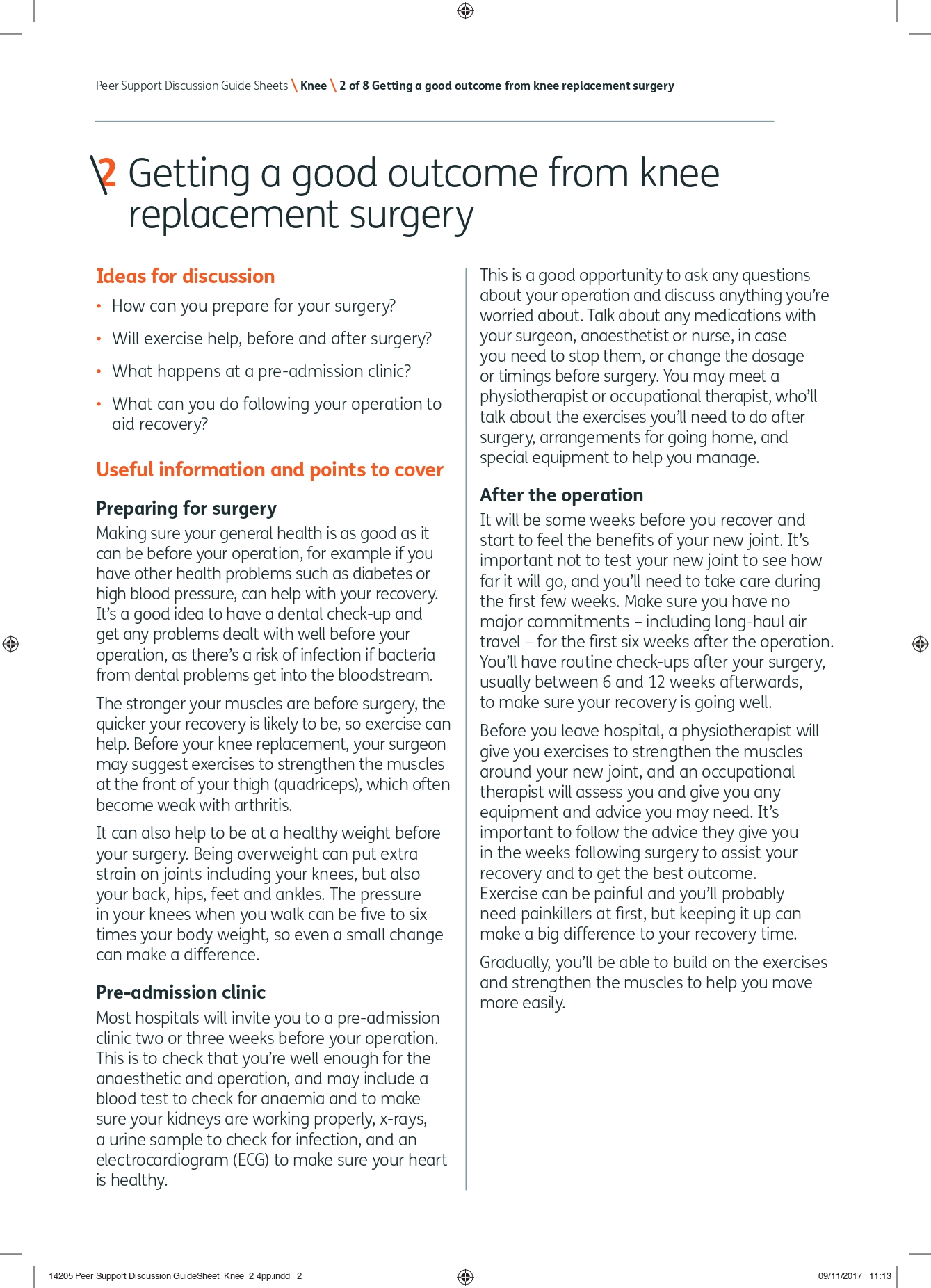


**Appendix 1**


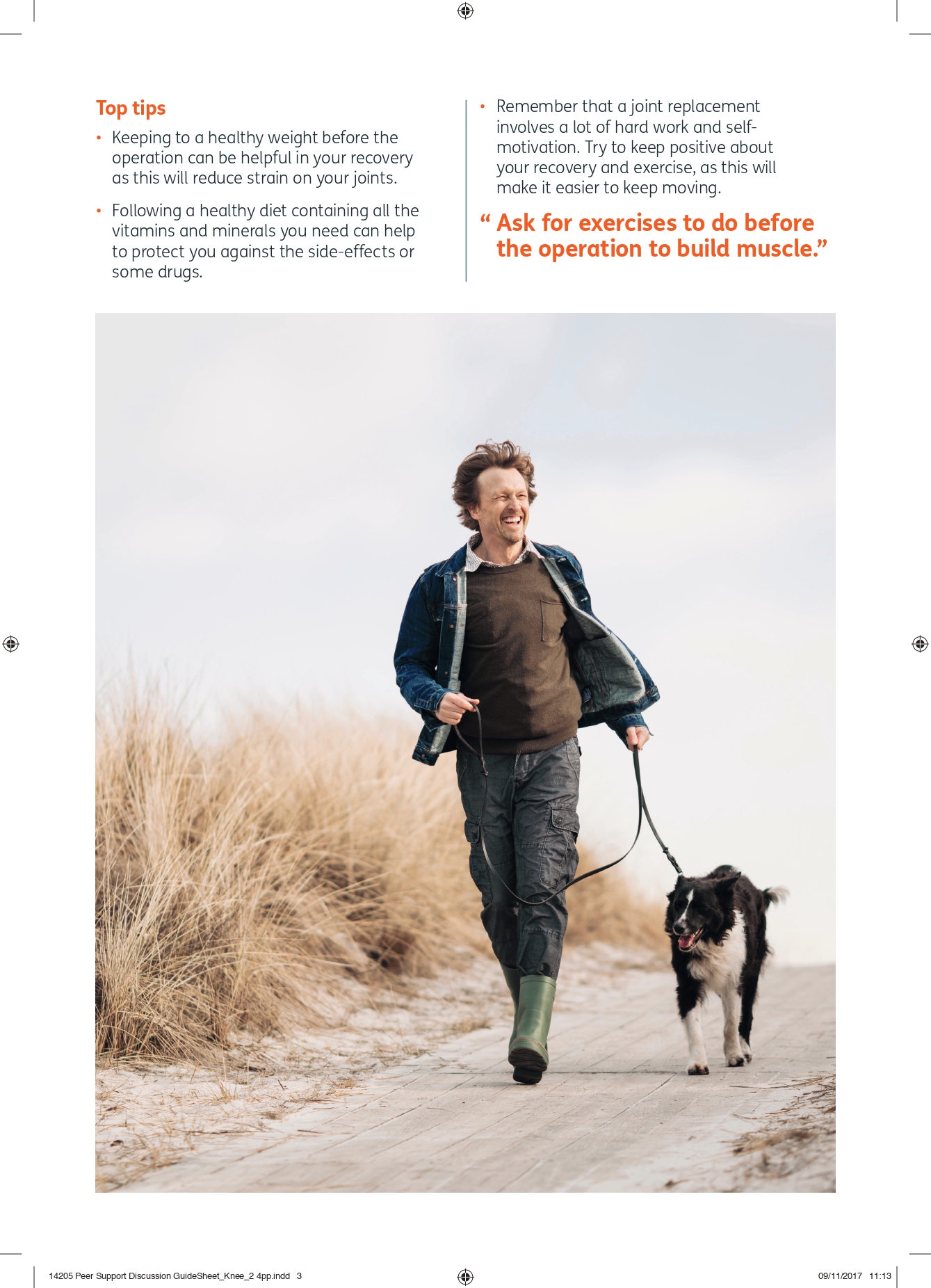

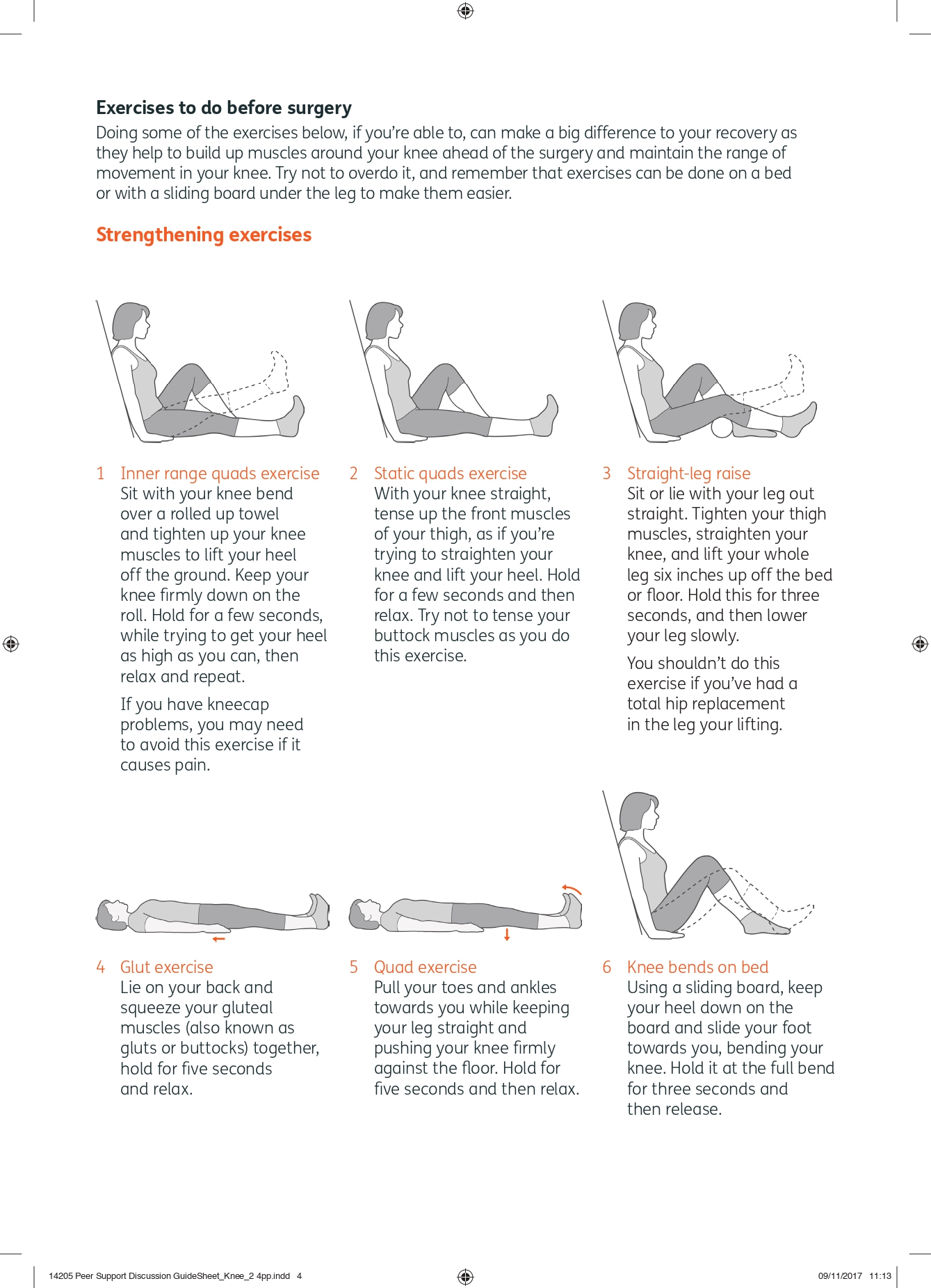

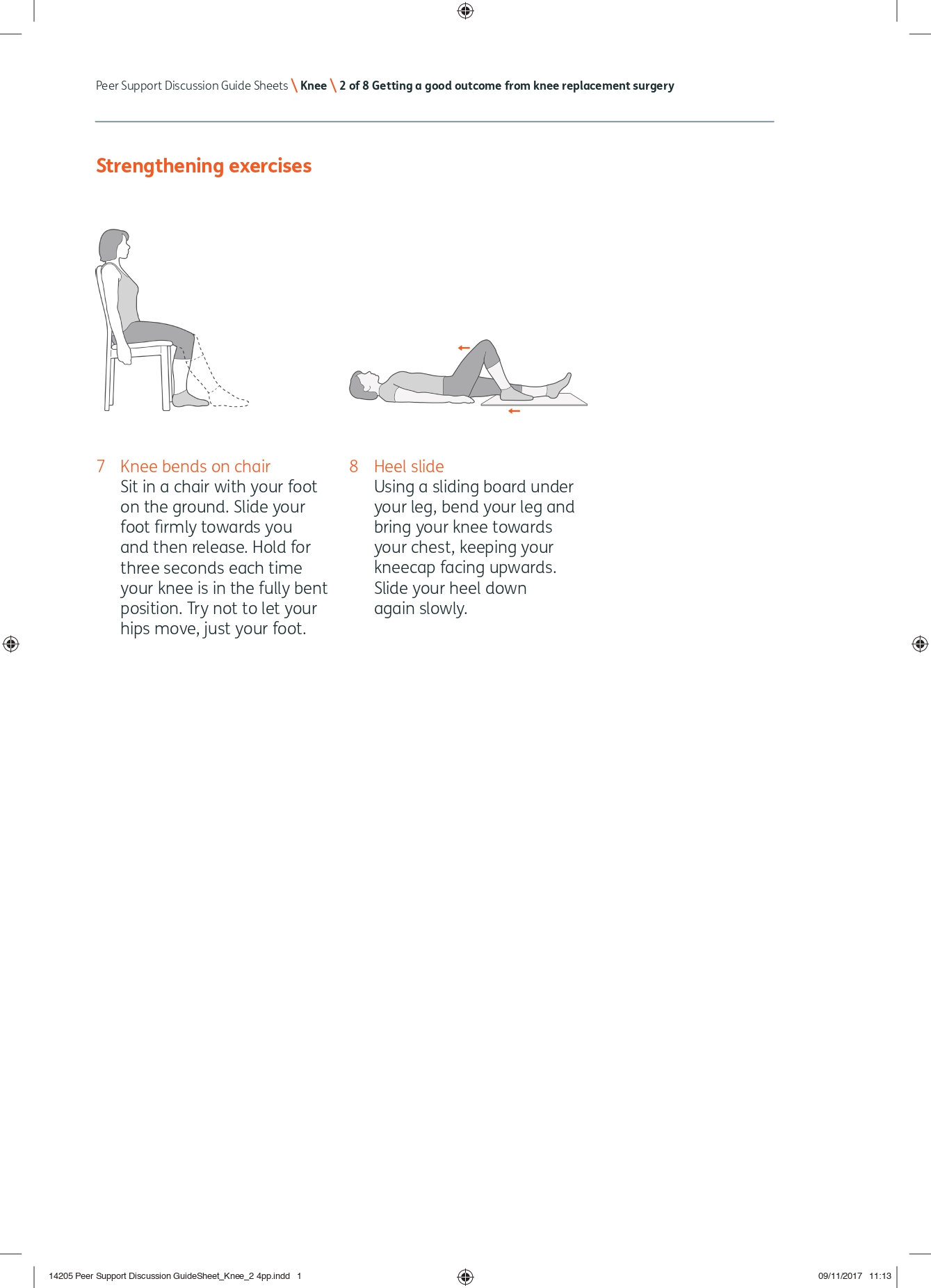

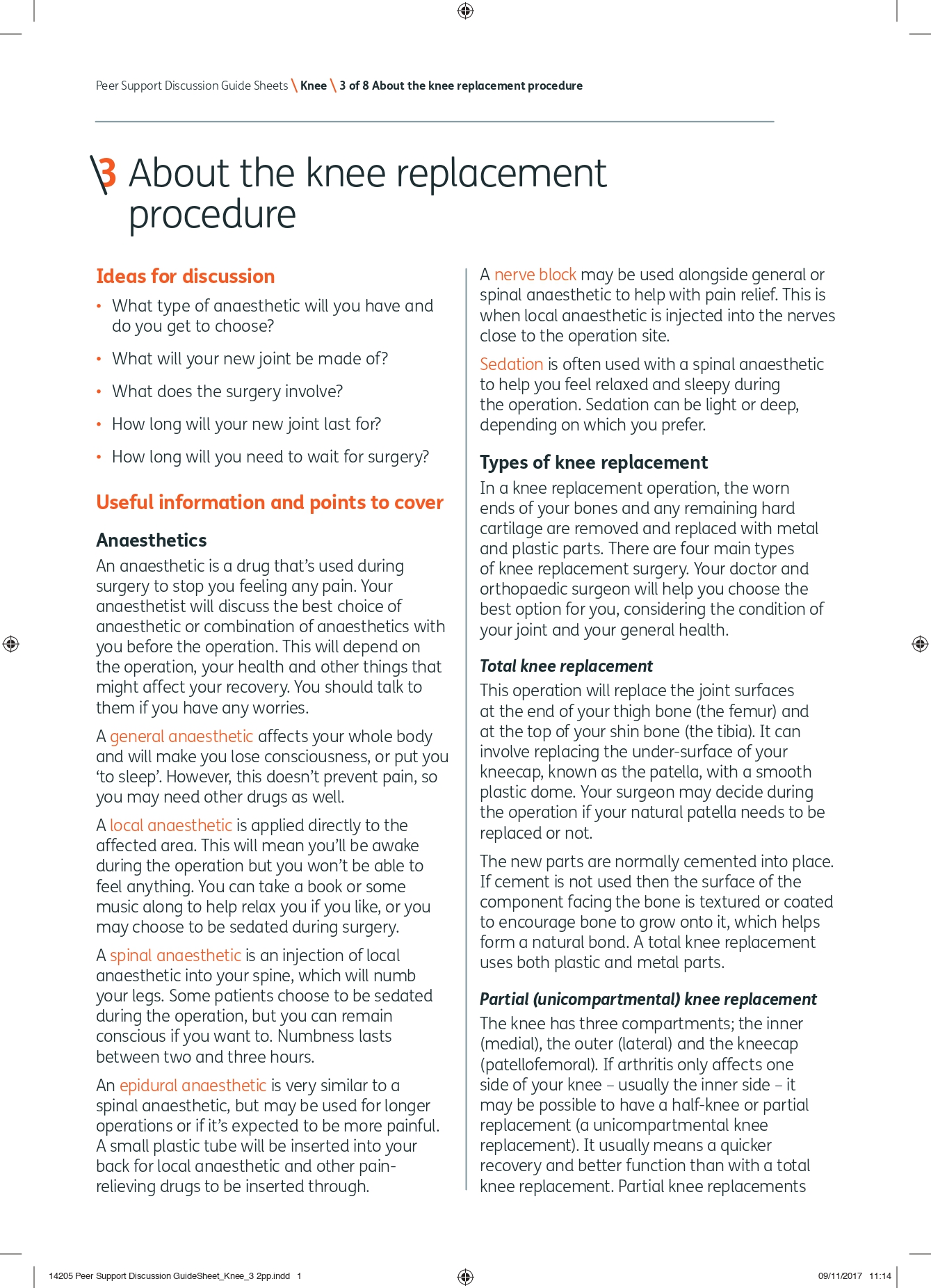

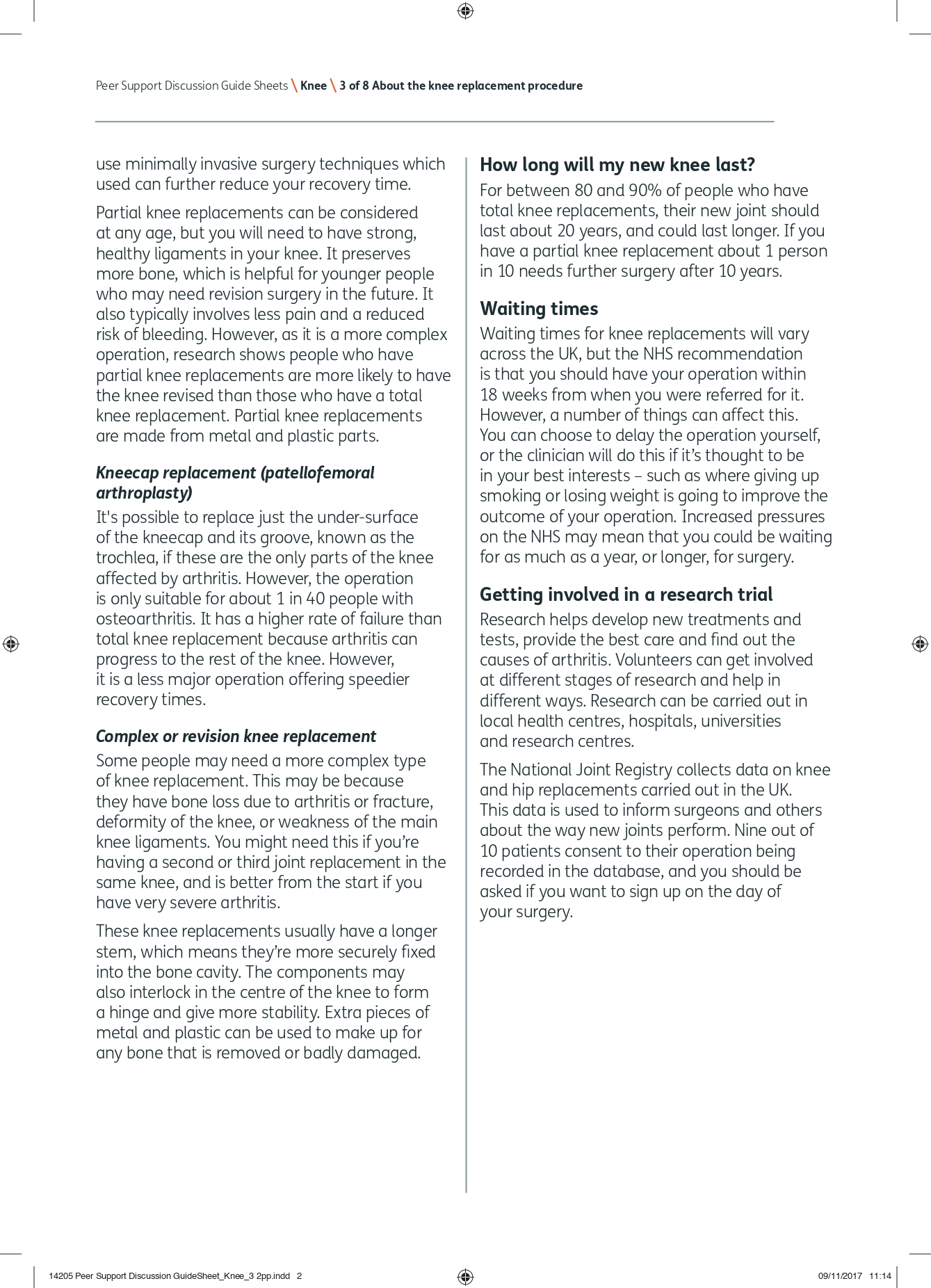

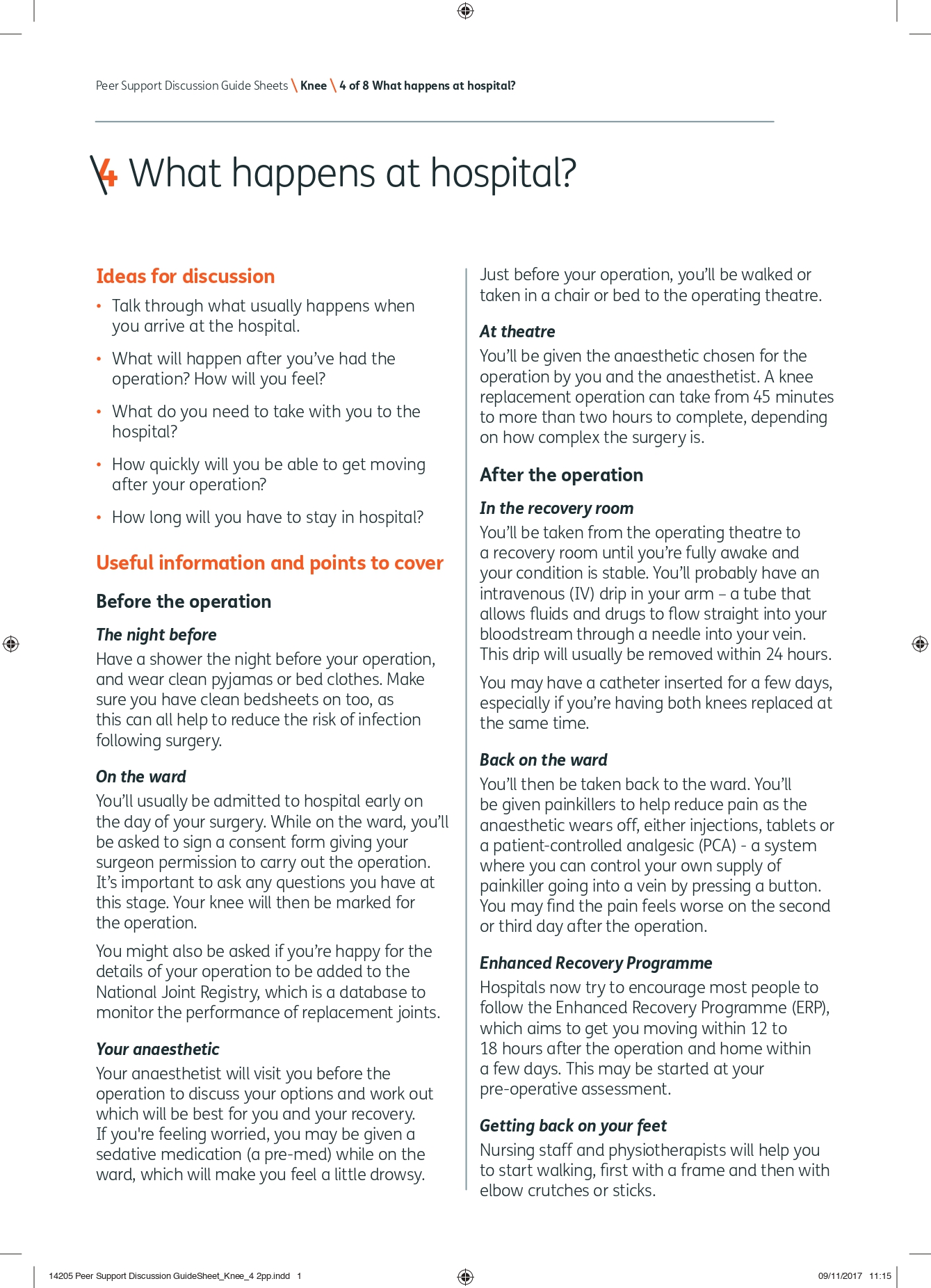

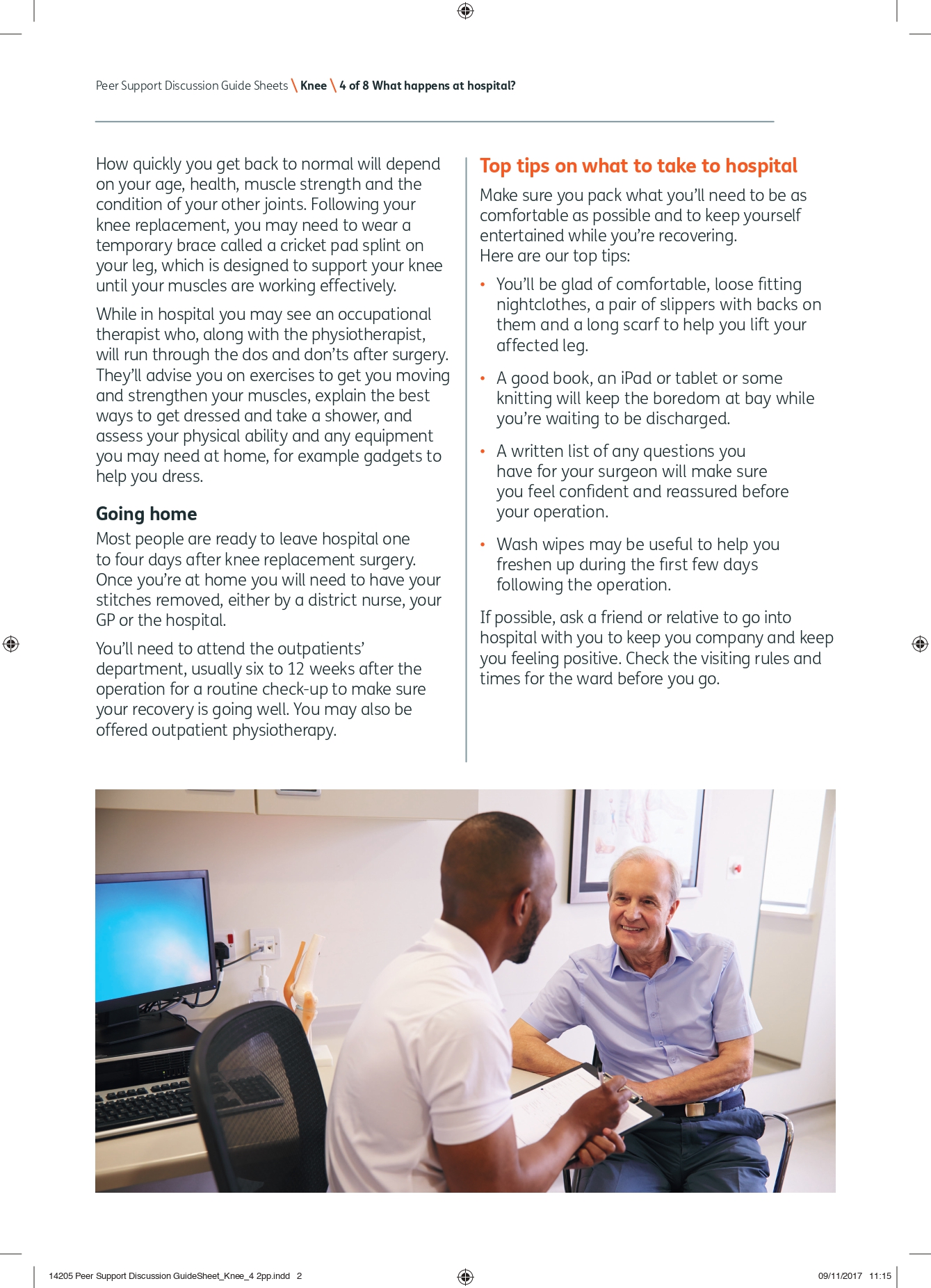

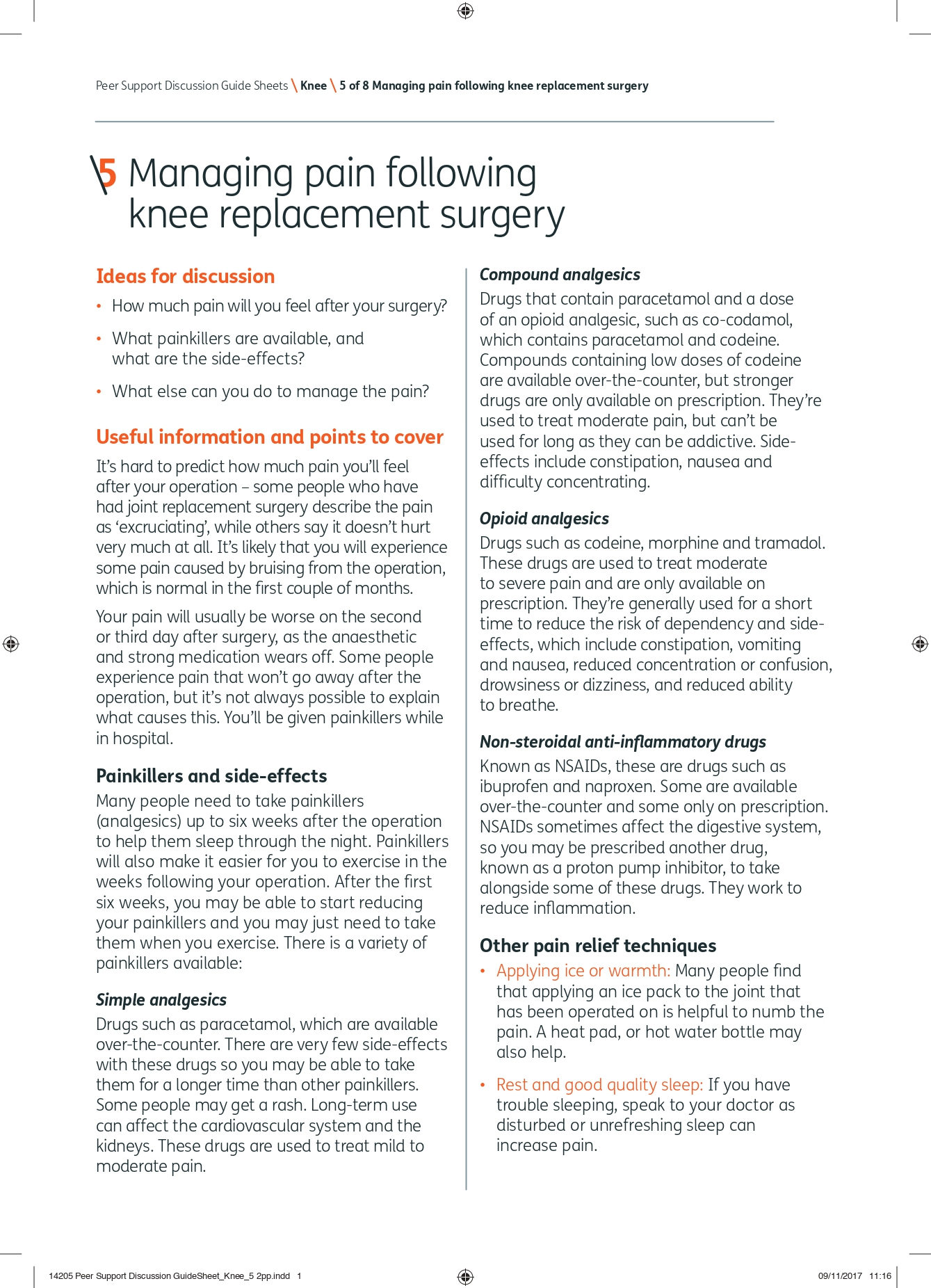

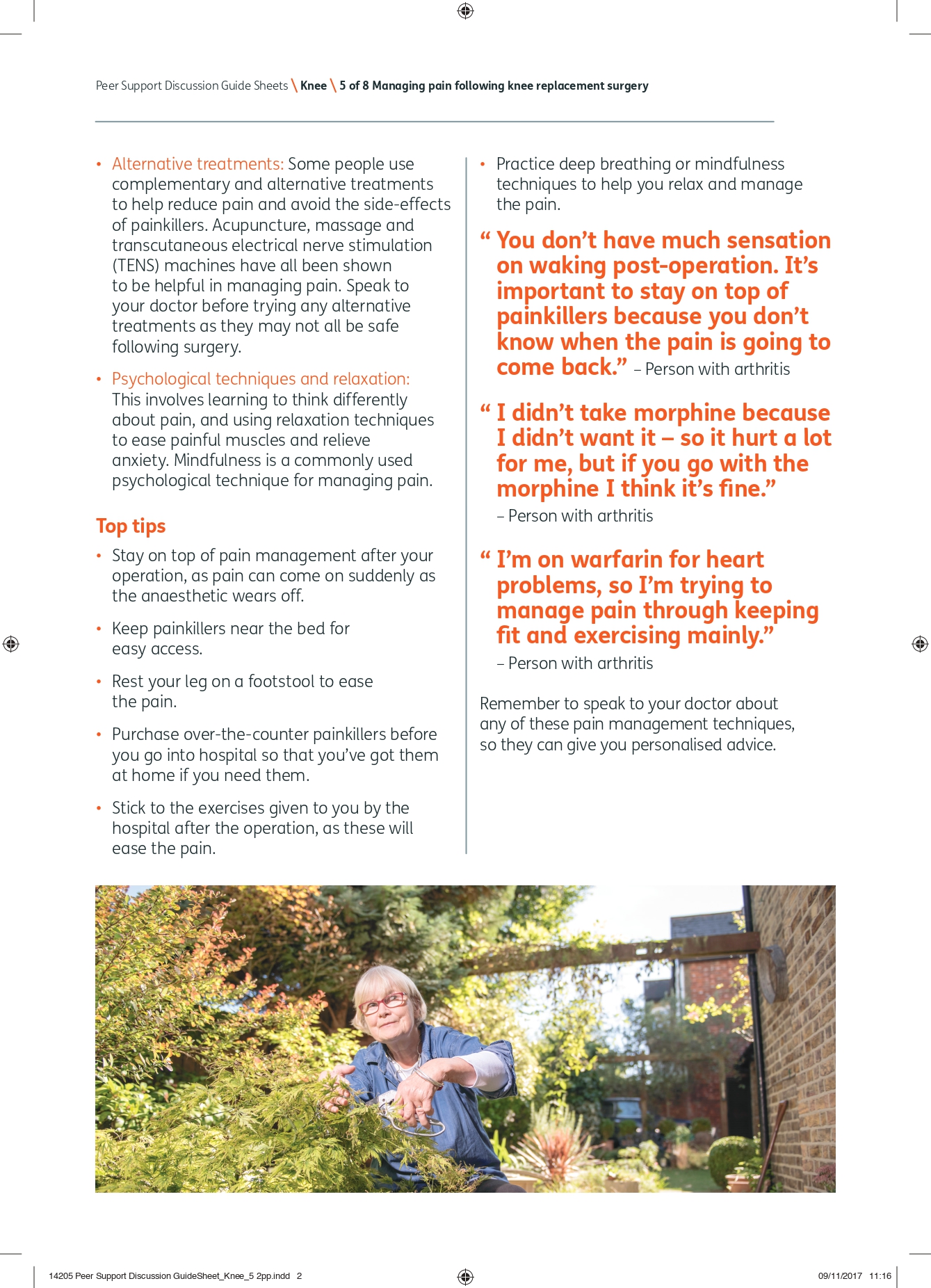

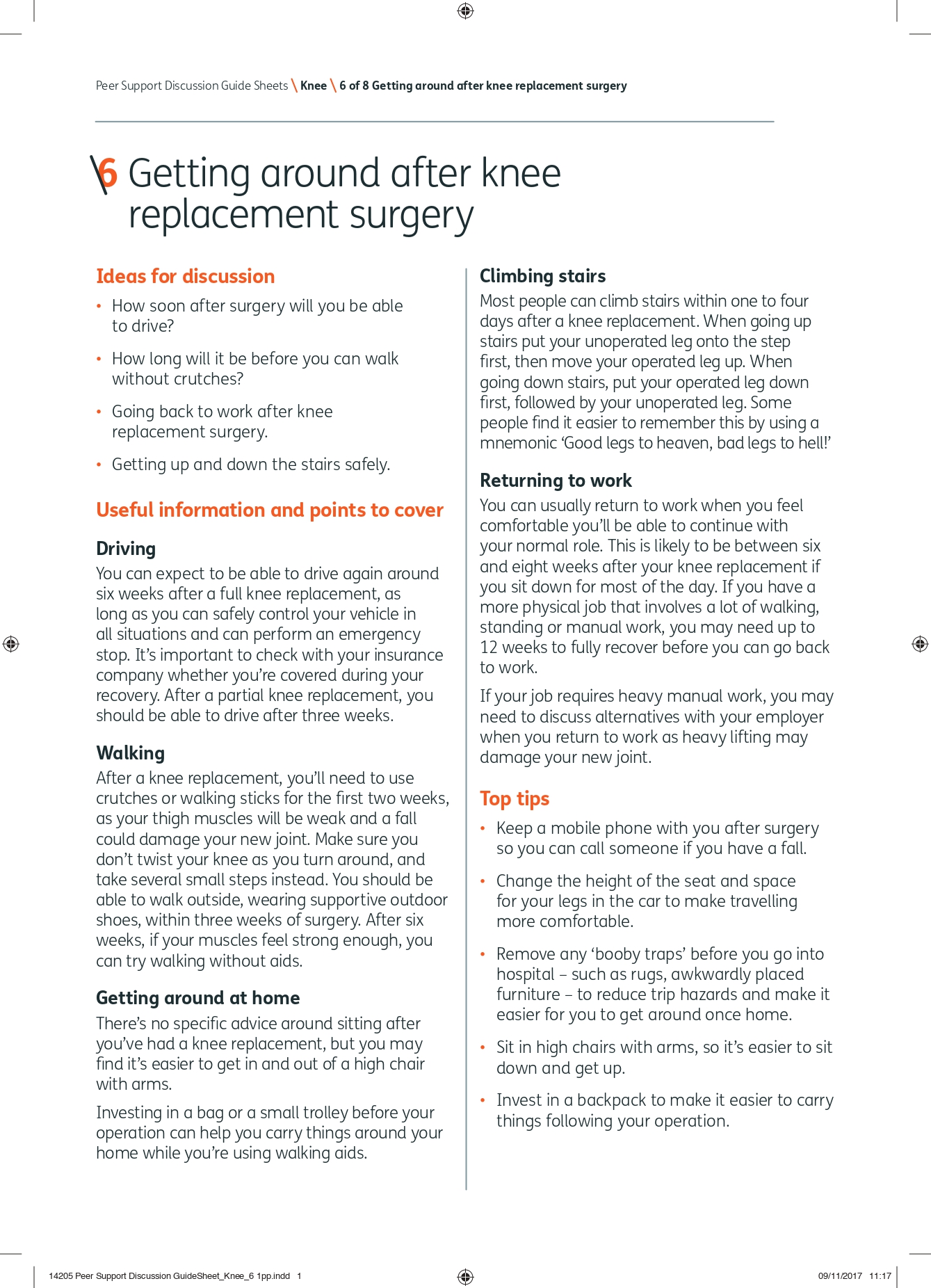

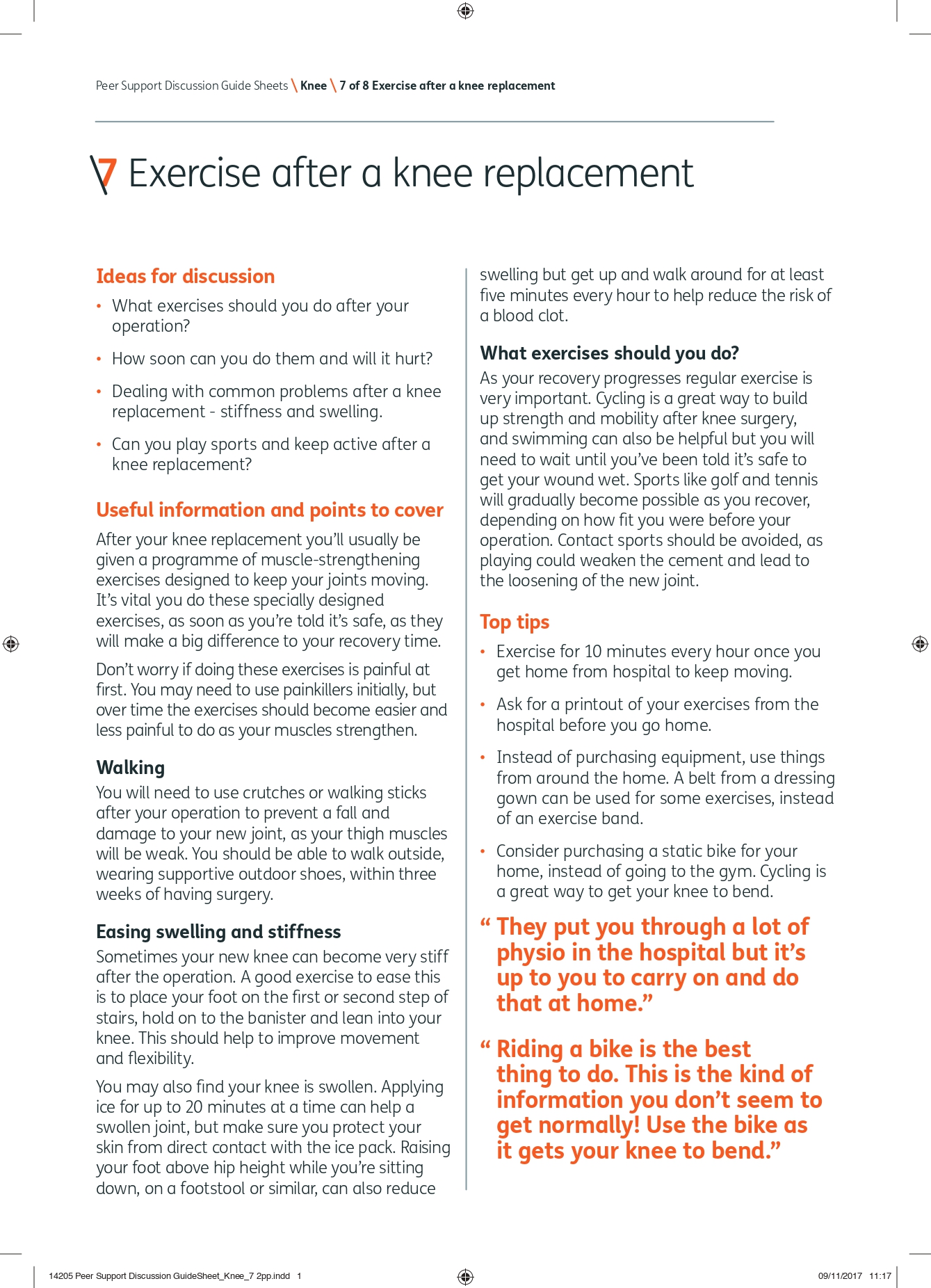

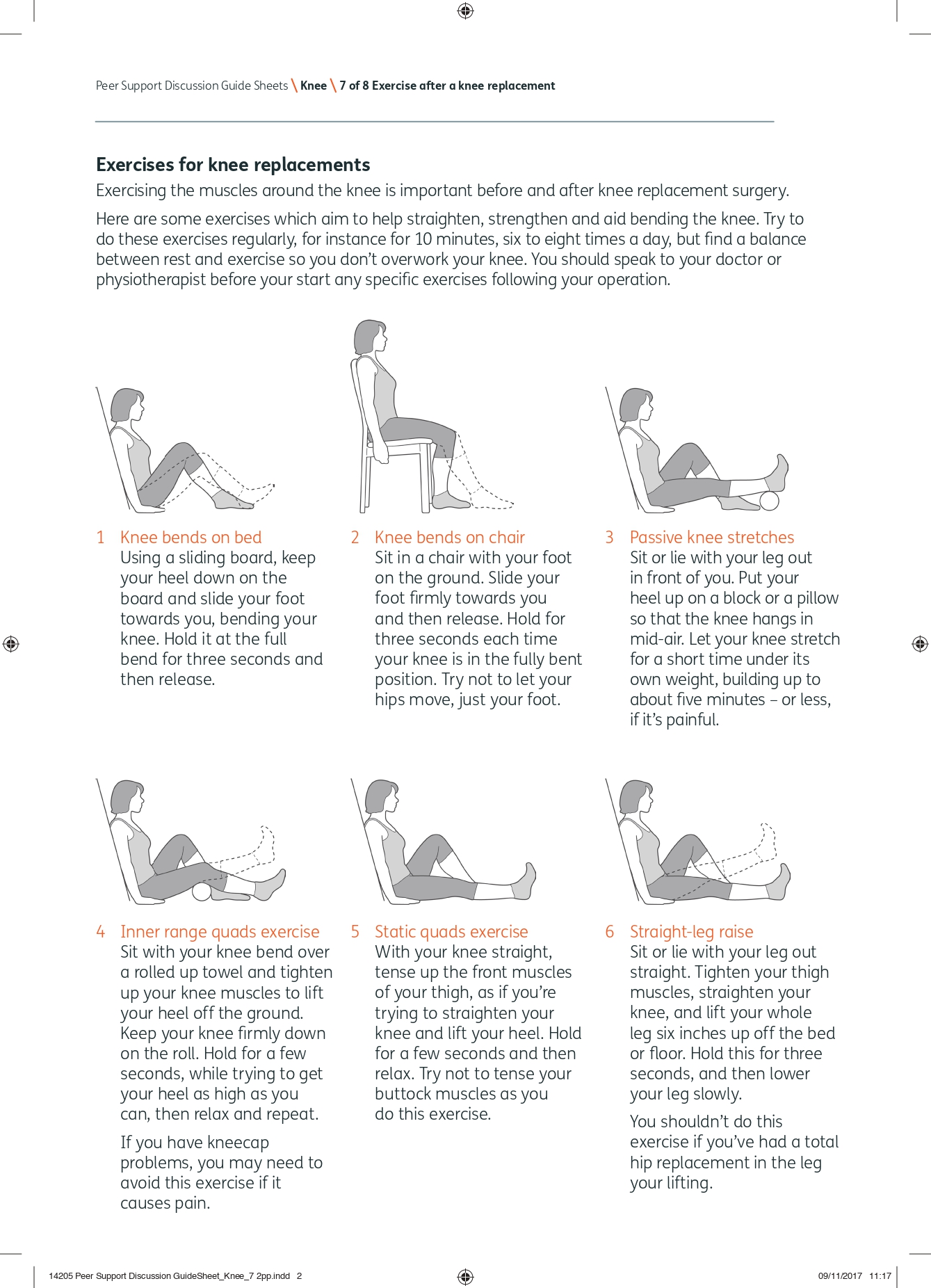

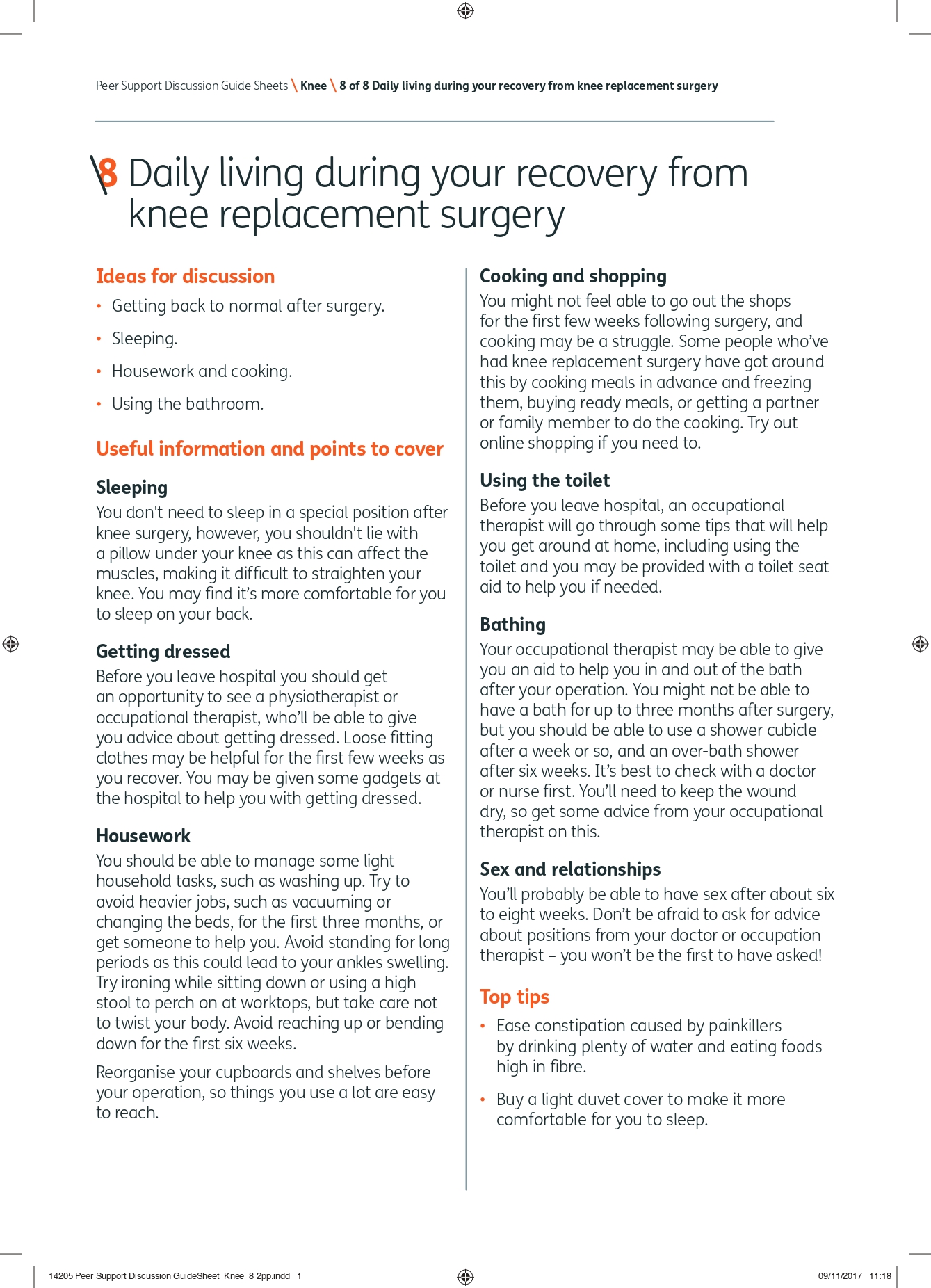

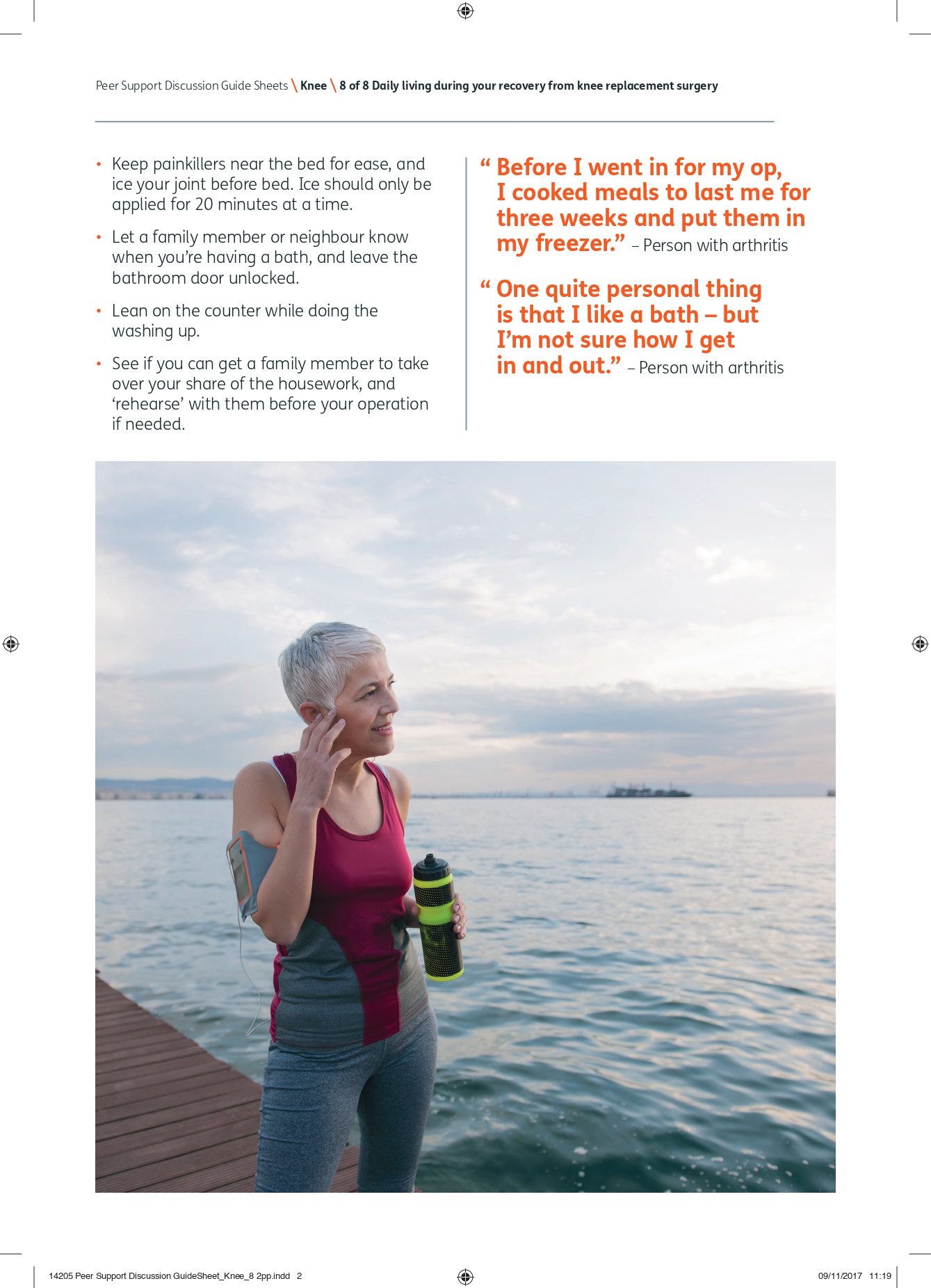

Supplement: Supplementary file 1 — Supporting File 1. [file JEO2-13-e70777-s002.docx]
