# Supplementary material for: Patient‐reported outcomes following participation in a preoperative peer support programme for total knee replacement: A prospective observational cohort study
Source: J Exp Orthop. 2026 Jun 4;13(2):e70777. doi: 10.1002/jeo2.70777 (PMC13288369; doi:10.1002/jeo2.70777)
Supplement: Supplementary file 2 — Supporting File 2. [file JEO2-13-e70777-s001.docx]

**Appendix 2**

The 10 statements included in the questionnaire are as follows:

- I am satisfied with the pain relief I achieve for arthritis / joint pain with my current treatment plan / medication
- I often feel like I cannot do anything myself to lessen the impact of arthritis on my life (reversed)
- I feel there is someone who understands what I'm going through
- I have people around me who can support me
- I am able to manage my health in ways that work for me
- I feel anxious / worried about my operation (reversed)
- I have the information I need to know what to expect from the operation
- I have the information I need to support my recovery after the operation
- I have support from people who are going through the same experience
- Overall, how would you rate the impact of arthritis / joint pain on your day-to-day life? 1 not at all, 10 very severe (reversed)
